# Neuropilin 1 Involvement in Choroidal and Retinal Neovascularisation

**DOI:** 10.1371/journal.pone.0169865

**Published:** 2017-01-20

**Authors:** Patricia Fernández-Robredo, Senthil Selvam, Michael B. Powner, Dawn A. Sim, Marcus Fruttiger

**Affiliations:** 1 UCL Institute of Ophthalmology, University College London, London, United Kingdom; 2 Experimental Ophthalmology Laboratory, School of Medicine, University of Navarra, IdiSNA, Navarra Institute for Health Research, Pamplona, Spain; 3 Division of Optometry and Visual Science, School of Health Sciences, City University London, London, United Kingdom; 4 NIHR Biomedical Research Centre for Ophthalmology, Moorfields Eye Hospital NHS Foundation Trust, London, United Kingdom; University of Tasmania, AUSTRALIA

## Abstract

**Purpose:**

Inhibiting VEGF is the gold standard treatment for neovascular age-related macular degeneration (AMD). It is also effective in preventing retinal oedema and neovascularisation (NV) in diabetic retinopathy (DR) and retinal vein occlusions (RVO). Neuropilin 1 (Nrp1) is a co-receptor for VEGF and many other growth factors, and therefore a possible alternative drug target in intra ocular neovascular disease. Here we assessed choroidal and retinal NV in an inducible, endothelial specific knock out model for Nrp1.

**Methods:**

Crossing Nrp1 floxed mice with Pdgfb-CreERT2 mice produced tamoxifen-inducible, endothelial specific Nrp1 knock out mice (Nrp1^ΔEC^) and Cre-negative, control littermates. Cre-recombinase activity was confirmed in the Ai3(RCL-EYFP) reporter strain. Animals were subjected to laser-induced CNV (532 nm) and spectral domain-optical coherence tomography (SD-OCT) was performed immediately after laser and at day 7. Fluorescein angiography (FA) evaluated leakage and postmortem lectin staining in flat mounted RPE/choroid complexes was also used to measure CNV. Furthermore, retinal neovascularisation in the oxygen induced retinopathy (OIR) model was assessed by immunohistochemistry in retinal flatmounts.

**Results:**

In vivo FA, OCT and post-mortem lectin staining showed a statistically significant reduction in leakage (p<0.05), CNV volume (p<0.05) and CNV area (p<0.05) in the Nrp1^ΔEC^ mice compared to their Cre-negative littermates. Also the OIR model showed reduced retinal NV in the mutant animals compared to wild types (p<0.001).

**Conclusion:**

We have demonstrated reduced choroidal and retinal NV in animals that lack endothelial Nrp1, confirming a role of Nrp1 in those processes. Therefore, Nrp1 may be a promising drug target for neovascular diseases in the eye.

## Introduction

A final common complication for multiple retinal diseases, such as age related macular degeneration (AMD), diabetic retinopathy (DR) and retinal vein occlusions (RVO) is the growth of abnormal neovascular blood vessels (neovascularisation) with increased permeability that produce fluid leakage in the macula [1,2], which can lead to vision loss. Neovascularization may originate from choroidal blood vessels (CNV) invading Bruch’s membrane and the subretinal space, or from the retinal vasculature. In both cases the abnormal angiogenesis is often driven by excessive production of vascular endothelial growth factor (VEGF), and inhibiting VEGF is the current gold standard in the treatment of neovascular AMD [3]. Moreover, intravitreal administration of VEGF blockers (Ranibizumab, Aflibercept and off-label Bevacizumab) is also effective in preventing retinal oedema and neovascularisation in DR and RVO [4,5]. However, there are limitations. Although VEGF blockage can halt pathological angiogenesis, reduce vessel leakage and cause regression of existing vessels [6], it does not address the causes that drive the disease and requires monthly/bimonthly intravitreal injections. Furthermore, they are only beneficial in subsets of patients [7]. There is also the concern that VEGF is a neuronal survival factor and sustained blocking of VEGF may have undesirable side effects [1,2]. It is therefore important to explore alternative molecular targets for the development of therapies blocking retinal angiogenesis.

Besides VEGF, there are several other factors known to regulate angiogenesis, such as transforming growth factor beta (e.g. TGFB1), fibroblast growth factors (e.g. FGF2), semaphorins (e.g. SEMA3E) and angiopoietins (e.g. ANGPT2) [8–12]. Neuropilin 1 (NRP1) functions as a co-receptor for several of these ligands (e.g. VEGF, TGFB1, FGF2 and semaphorins) [13–16] and might therefore be a promising target to interfere with multiple signalling pathways simultaneously to overcome VEGF-resistance. In this context it is noteworthy that genetic variation in Nrp1 seems to influence treatment responses to anti-VEGF therapy in neovascular AMD [17].

The general importance of NRP1 in angiogenesis has previously been demonstrated in numerous studies. For instance, Nrp1 null mutations in mice cause serious vascular abnormalities [18,19] and deficient endothelial tip cell function in sprouting angiogenesis [20,21] during embryogenesis. Several studies on cultured endothelial cells showed an involvement of Nrp1 in VEGF—VEGFR2 signalling [22–24]. However, mice harbouring a non-VEGF binding mutant form of Nrp1 develop normally [25], suggesting that the essential function of Nrp1 in sprouting angiogenesis depends on ligands other than VEGF. In fact, two recent studies showed that Nrp1 critically regulates TGFB/BMP signalling in central nervous system (CNS) vascular development [26,27].

Since sprouting angiogenesis not only contributes to vessel growth during embryogenesis but also to neovascularisation in adult eye pathologies, we assessed the role of Nrp1 in choroidal and retinal neovascularisation in endothelium specific, inducible Nrp1 knockout (Nrp1^ΔEC^) mice. CNV in mice was triggered by laser lesions, which is a widely accepted and reproducible model that mimics many features of CNV occurring in the wet form of AMD. Although the acute laser injury does not mimic the chronic disease condition in humans, the model is useful for the investigation of the cellular and molecular mechanisms that drive CNV. We also used the oxygen induced retinopathy (OIR) model [28,29] to study effects on neovascularisation in the retinal vasculature.

## Animals, Materials and Methods

### Animals

All animals were handled in accordance with the United Kingdom (UK) Animals (Scientific Procedures) Act 1986 and all experiments were covered by a project license approved by the UK Home Office (PPL7157) and the University College of London (UCL) Institute of Ophthalmology (IOO) Ethics Sub-Committee. Due to lethality of Nrp1 knock out animals it was necessary to use an inducible Cre-lox approach. We therefore crossed a tamoxifen-inducible, endothelial cell specific Cre strain (*Pdgfb-CreER* mice) [30] with Nrp1 floxed [18] mice, creating endothelial specific Nrp1 knock out mice (Nrp1^ΔEC^). Cre negative littermates were used as controls. Cre recombinase was induced in adult animals (6–8 weeks old) by injecting 200 μl of tamoxifen (15 mg/ml in soybean oil; Sigma-Aldrich) i.p. daily for 4 days before laser application. To assess Cre recombinase activity the reporter strain Ai3(RCL-EYFP) was used (conditionally expressing EYFP from a CAG promoter in the ROSA locus).

### Laser CNV model

Mice were anesthetized with a mixture (i.p.) of Ketamine (Ketaset, Lyon, France, 75 mg/kg) and xylazine (Domitor 2%, 10mg/kg; Bayer Animal Health, Leverkusen, Germany). Pupils were dilated with a mixture of tropicamide 0.5% (Bausch and Lomb) and phenylephrine 1% (Bausch and Lomb) and a diode laser (wavelength 532 nm, Micron III, Phoenix Research, USA) with a power of 120 mW, an exposure time of 0.1 seconds and a spot size of 50 μm was used to induce 3–4 CNV lesions in the retina. Laser lesions were applied approximately two optic discs from the optic nerve, while avoiding major blood vessels. Vaporisation bubble formation confirmed the rupture of Bruch’s Membrane [31] and those spots that did not result in the formation of a bubble were excluded. Laser photocoagulation sites that developed CNV were analysed 1 week after laser application.

### OIR model

Litters were placed in 75% oxygen chamber fitted with an oxygen controller (PROOX 110; Reming Bioinstruments Co., Redfield, USA) from P7-12 and kept a room air until P17 [28]. Cre recombinase was activated by i.p. injection of 4-hydroxy-tamoxifen (OHT) at P11 (in the OIR model, 5μg/pup) or at P3 (2μg/pup to test Nrp1 deletion efficiency). Eyes were then processed for wholemount immunohistochemistry as previously described [30], using antibodies against Nrp1 (1:200, R&D systems, AF566), collagen IV (1:200, AbD Serotec, 2150–1470), conjugated α-smooth muscle actin (ASMA, 1:200, Cy3-conjugated, Sigma Aldrich, C6198) and FITC-conjugated isolectin B4 (1:200, Vector Labs). Wholemounts of retinas were imaged and analysed according to published protocols [32]. This involved manually tracing and quantifying the neovascular and avascular areas with ImageJ.

### In vivo imaging

OCT horizontal scans (400x400x1024 voxels), centred on the optic nerve head, were obtained from both eyes of the mice using an R2200 UHR SD-OCT scanner (Bioptigen Inc., Morrisville, NC, USA) on day 0 (0.5–1 hour after laser application) to confirm rupture of Bruch’s membrane and on day 7 to measure the volume of CNV lesions. Images were captured by In Vivo Vue software (Bioptigen Inc.) and the obtained scans were converted to avi-files in order to measure the volume with Fiji Software V1.48q (a distribution of ImageJ). Measurements were made by two independent investigators.

A scanning laser ophthalmoscope (Spectralis^™^ HRA, Heidelberg Engineering, Heidelberg, Germany) with a 55° field of view lens was used to assess EYFP expression in Cre reporter mice. One week after laser application, animals were subjected to fluorescein angiographic evaluation. Fluorescein angiograms were obtained using a retinal imaging microscope (Micron III, Phoenix Research, USA) was used. Briefly, after pupil dilation anaesthetised animals were i.p. injected with 100 μl of 10% sodium fluorescein and images were obtained after 1–2 min (early leakage) and 6–7 min (late leakage). Area of leakage was measured by two trained independent investigators using Fiji Software V1.48q (a distribution of ImageJ).

### Post-mortem histology

After sacrificing the animals, eyes were enucleated and immediately fixed in 4% paraformaldehyde for 10–15 minutes. Retinas and scleras (with choroid and RPE attached) were dissected separately and flattened by making radial cuts. In order to assess recombination efficiency in the reporter strain, YFP fluorescence was imaged by confocal microscope (LSM710, Carl Zeiss, Meditec, Dublin, CA) directly in wholemounts of RPE-choroid complexes and in cryosections. Whole mounts and cryosections were also subjected to immunohistochemistry to visualise vessels and inflammatory cells using biotinylated isolectin (1:200, IB4, Vector Labs), CD11b (1:200, Abcam, ab64347), Nrp1 (1:200, R&D systems, AF566), Hoechst 33258 (1:1000, Sigma-Aldrich), streptavidin coupled to Alexa Fluor^®^ 594 (1:250, Life Technologies) and chicken anti-rat IgG Alexa Fluor^®^ 647 conjugated (1:200, Life Technologies). CNV lesion areas were captured by confocal microscopy and measured by two independent and trained investigators by using Fiji Software V1.48q (a distribution of ImageJ).

### Statistical analysis

Only laser lesions that developed CNV were included in the statistical analysis. Exclusion criteria were the absence of a visible vaporisation bubble at the time of laser application, no evidence of BM rupture (as observed by OCT immediately after laser application) and the presence of haemorrhage (larger than the laser spot). A non-parametric Kruskal-Wallis test was performed to assess differences between control and Nrp1^ΔEC^ animals, using SPSS statistics software (SPSS Inc., Chicago, IL). N-numbers refer to the number of mice throughout the manuscript.

## Results

### Confirmation of Cre recombinase activity

**Fig 1:**
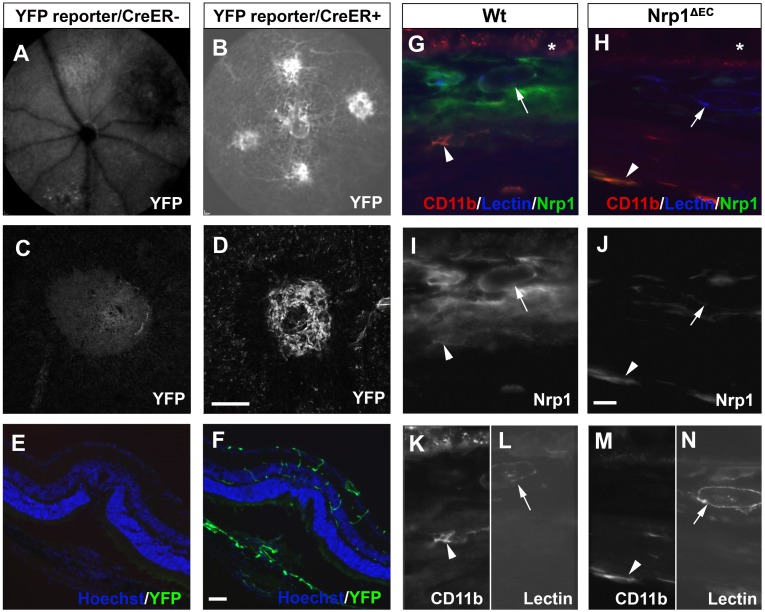
Confirmation of Cre recombinase activity and Nrp1 deletion in the choroidal vasculature. A YFP reporter strain (A-F) was used to demonstrate recombination activity in animals carrying the tamoxifen-inducible transgene Pdgfb-CreER (B, D, F). CreER negative animals were used as a negative control (A, C, E). In vivo imaging (A, B) using the fluorescein channel in a Spectralis ophthalmoscope focused on the choroid showed YFP expression in most vessels, including laser-induced neovascular foci. Microscopy on postmortem tissue also demonstrates pronounced YFP fluorescence in laser-induced CNV lesions in choroidal flatmounts (C, D), and in cross sections (E, F) showing Hoechst staining (blue) and YFP fluorescence (green). Microscopy on cross sections from the choroid from Nrp1^ΔEC^ mice (H, J, M, N) or wild type controls (G, I, K, L) was used to demonstrate deletion of Nrp1 from endothelial cells (white arrows) by immunohistochemistry against Nrp1 (green in G, H, white in I, J). Nrp1 expressed by microglia (arrowheads), stained with anti-CD11b (red in G, H, white in K, M), was not targeted (I, J). Asterisks in G, H indicate autofluorescent RPR. Scale bars in D, F are 100 μm and in J 10 μm.

In order to confirm that the Pdgfb-CreER strain can drive Cre recombination in the adult choroidal vasculature, the Ai3(RCL-EYFP) reporter strain was used. One week after laser injury, *in vivo* laser scanning ophthalmoscopy revealed pronounced fluorescence in most vessels and in laser lesions in animals with the Cre allele but not in Cre negative controls (Fig 1A and 1B). Enhanced yellow fluorescent protein (EYFP) fluorescence was confirmed by confocal microscopy on *post-mortem* choroid whole mount preparations (Fig 1C and 1D) and on cryosections through laser lesions (Fig 1E and 1F). This demonstrated that the Pdgfb-CreER strain effectively causes recombination of floxed alleles in the adult choroidal vasculature. No differences in vessel growth or health were observed between CreER positive or negative animals (neither during development nor in the adult) in the absence of tamoxifen induction, suggesting the CreER transgene did not have any off-target effects.

The Pdgfb-CreER strain was crossed with a Nrp1-floxed strain to produce offspring with or without the CreER transgene in a homozygous Nrp1-floxed background. Upon tamoxifen treatment, Nrp1 protein was depleted in choroidal endothelial cells (arrows in Fig 1) in CreER positive (Nrp1^ΔEC^) but not in CreER negative (Wt) animals (Fig 1G–1J). CD11b positive microglial cells (arrowheads in Fig 1) maintained Nrp1 expression, irrespective of genotype (Fig 1K–1N), confirming the endothelial specificity of the Nrp1 depletion.

### CNV in vivo volume is reduced in Nrp1^ΔEC^ animals

**Fig 2:**
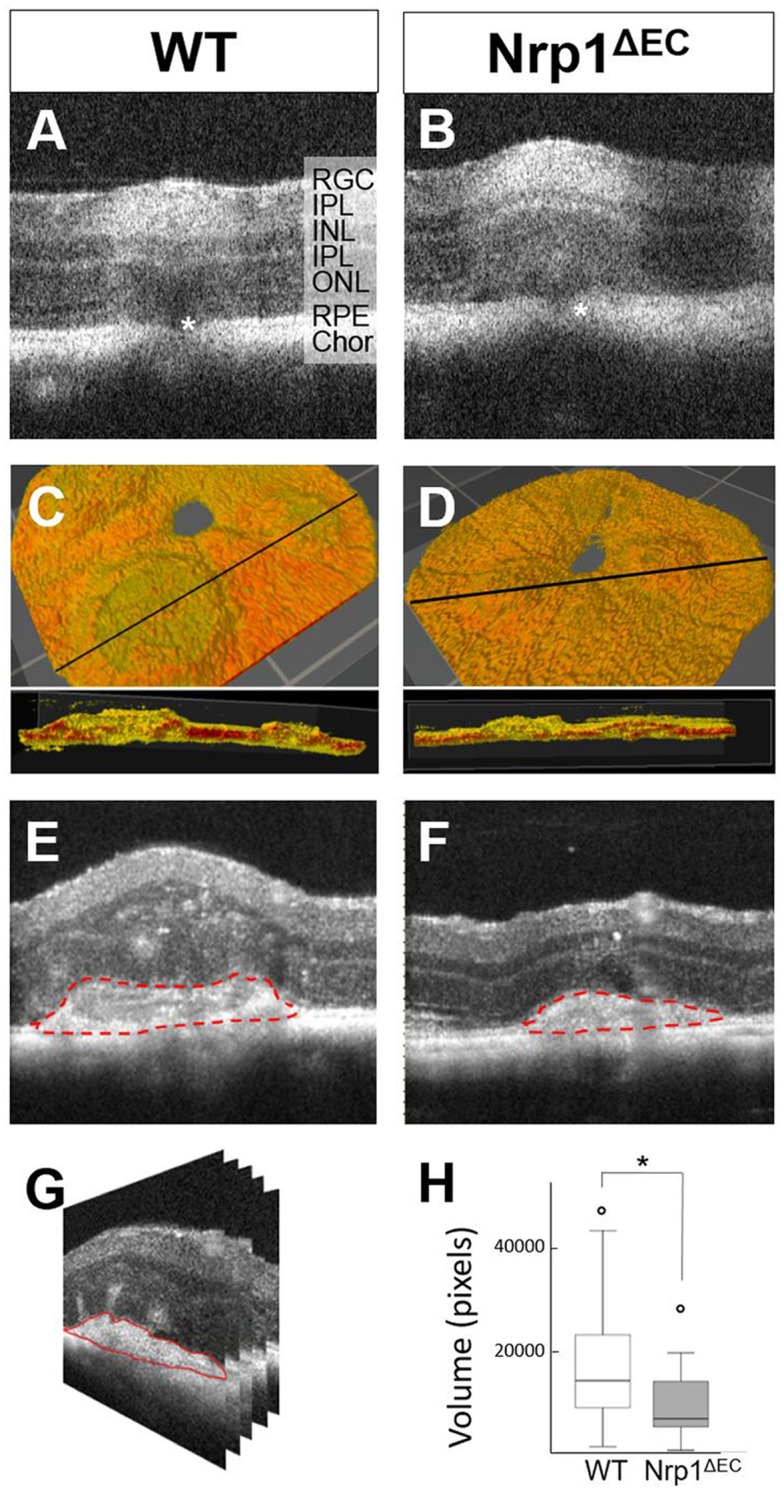
Laser-induced CNV assessed by OCT. *In vivo* OCT images obtained immediately after laser burns were applied, demonstrate Bruch’s membrane ruptures (asterisks in A, B) in WT (left column) and Nrp1^ΔEC^ (right column) mice. One week after laser-induction, CNV lesions were imaged and quantified (C-H). The top panels in C, D represent 3D reconstructions of the RPE and CNV lesion surfaces. The bottom panels in C, D are cross sections along the black lines in the panels above (red corresponds to white the black and white images and indicates the RPE. Dotted red lines in E, F indicate the lesion borders measured, which were reconstructed into 3D volumes (G) with a distance of 14 μm between each frame. CNV volume measurement box-plots show significant volume reduction in Nrp1^ΔEC^ compared to WT mice (P<0.05; n = 7). RGC: Retinal Ganglion Cell; IPL: Inner Plexiform Layer; INL: Inner Nuclear Layer; OPL: Outer Plexiform Layer; ONL: Outer Nuclear Layer; RPE: Retinal Pigment Epithelium; Chor: Choroid.

To assess the effects of vascular Nrp1 depletion on neovascularisation in the retina, we utilised the laser-induced CNV model. OCT was used to measure CNV lesions *in vivo*. Firstly, rupture of Bruch’s membrane was confirmed immediately after the laser lesions were applied (Fig 2A and 2B). Then, 7 days later the laser lesions were re-examined using volume scans (Fig 2C and 2D). The volumes of CNVs within the lesions were measured by assessing the area of the CNVs (red stippled outline in Fig 2E and 2F) in each b-scan (Fig 2G). This showed a statistically significant (p = 0.018) reduction in CNV volume in transgenic animals compared to wild type mice (Fig 2H).

### Reduced leakage and CNV areas in Nrp1^ΔEC^ animals

**Fig 3:**
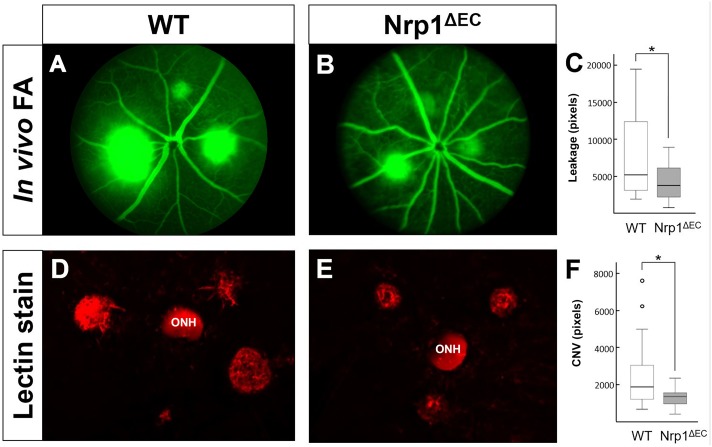
Laser-induced CNV assessed by fluorescein angiography and wholemount staining. In vivo FA images (A and B) and quantification of fluorescein leakage areas (C) one week after CNV induction showed stronger leakage in wild type (A) compared to Nrp1^ΔEC^ (B) mice (p<0.05; n = 5). Postmortem lectin staining (D and E) and CNV area quantification (F) one week after CNV induction also showed more pronounced pathology in wild type animals (p<0.05; n = 7). ONH indicates the optic nerve head.

The effects of endothelial Nrp1 deletion on CNV growth were also evaluated by fluorescein angiography (FA, Fig 3A and 3B) and post-mortem histology (Fig 3D and 3E). Measuring the hyper fluorescent areas in FA images showed a reduction in the Nrp1^ΔEC^ animals (p = 0.026, Fig 3C). Similarly, CNV areas measured in *post-mortem* histology (using IB4 lectin staining), also revealed a statistically significant (p = 0.026) reduction in lesion size in Nrp1^ΔEC^ mice *versus* Cre negative control mice (Fig 3F), confirming the OCT volume measurements by two independent assessment methods.

### Reduced neovascularisation in the OIR model in Nrp1^ΔEC^ mice

**Fig 4:**
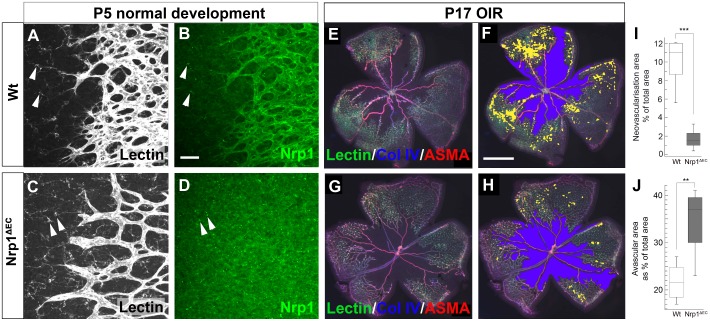
Loss of endothelial Nrp1 leads to reduced neovascularisation in the OIR model. Endothelial specific depletion of Nrp1 in the P5 retinal vasculature (OHT at P3) was confirmed by Nrp1 immunohistochemistry (A-D) on retinal whole mounts, visualising Nrp1 (green in B, D) and lectin staining (white in A, C). Nrp1 expressed by microglia (arrowheads in A-D) was not affected. Effects on OIR were assessed at P17 (OHT at P11) by immunohistochemistry on retinal whole mounts (E, G), showing lectin staining (green), collagen IV (blue) and alpha smooth muscle actin (red). Quantification of neovascularisation (painted yellow in F, H) and avascular areas (painted blue in F, H) showed reduced neovascularisation (I, p<0.001) and increased avascular areas (J, p = 0.01) in Nrp1^ΔEC^ animals compared to wild type animals (n = 6 Wt, n = 10 Nrp1^ΔEC^). Scale bars in B are 50 μm and in F 1000 μm.

In order to gain further insight about the role of Nrp1 in vascular pathologies in the retina, we also assessed the developing retinal vasculature. First, CreER efficiency was demonstrated in the retinal vasculature in Nrp1^ΔEC^ mice at the perinatal stage, by immunohistochemistry against Nrp1 on retinal wholemounts from postnatal day (P)5 pups that have been injected with OHT at P3 (Fig 4A–4D). This clearly confirmed endothelial cell specific Nrp1 depletion, whereas Nrp1 in microglial cells ahead of the developing plexus (arrowheads in Fig 4) was not affected. Furthermore, the growing edge of the developing retinal vasculature displayed less and thicker sprouting tips in Nrp1^ΔEC^ mice (Fig 1A–1C) as previously described [26].

We also assessed neovascular growth in the OIR model [28,29]. In this model perinatal mice are exposed to high oxygen levels, which depletes capillaries in the developing retinal vasculature, resulting in abnormal neovascular tuft formation upon return to room air. OHT was injected at P11 and neovascularisation was quantified at P17, which was significantly reduced in Nrp1^ΔEC^ animals compared to wild type litter mates (Fig 4E–4I). Interestingly, the remaining avascular area was larger in the mutant animals (Fig 4E–4H and 4J), suggesting that normal, regenerative vascular growth was also affected by Nrp1 deletion.

## Discussion

In this study we have demonstrated that Nrp1 mediated signalling is involved in neovascularisation during CNV development as well as during OIR, and could be a drug target in ocular neovascular diseases. CNV volume and fluorescein leakage were both reduced in the laser-induced CNV model in the absence of endothelial Nrp1, but the precise molecular mechanism of this effect is not understood yet. On the one hand VEGF is a well-established ligand for Nrp1 and is known to drive angiogenesis and vascular permeability during intraocular vascular disease [33,34]. On the other hand, Nrp1 is involved in aspects of TGFB superfamily signalling during sprouting angiogenesis [26,27]. More specifically, Nrp1 has been shown to be essential in endothelial tip-cells in developing brain blood vessels [20]. This may be based on its role in supressing the stalk-cell phenotype in endothelial tip-cells via the modulation of Alk1 and Alk5 mediated TGFB superfamily signalling [26]. In a pathological context, we have previously demonstrated that inhibition of TGFB can reduce CNV lesion size in the laser-induced CNV model [35–37]. This confirms that blocking of not only VEGF but also of other targets can be effective in preventing CNV. Therefore, targeting Nrp1 as a treatment strategy in ocular neovascular diseases might overcome anti-VEGF resistance.

In this report we used three different readout methods for the laser-induced CNV model: in vivo OCT, and FA, and postmortem histology. Although, each of the three methods assess slightly different aspects of CNV, all of them showed a clear effect caused by Nrp1 depletion. Postmortem histological analysis has the advantage that vascular elements and invading inflammatory cells can be identified separately and volume measurement of the CNV lesions can be obtained with high precision using confocal microscopy [38]. However, dissection and fixation artefacts might introduce noise into those volume measurements. In contrast, evaluating CNV lesions with OCT, measures the in vivo volume of CNV lesions and might be less affected by histological processing artefacts. Many authors have assessed CNVs in vivo based on FA images, but this approach does not take into account the height of the lesion and is probably, out of the three methods discussed here, the least reliable to evaluate CNV area. On the other hand, FA offers complementary data regarding vascular leakage, which the other two methods cannot assess. In summary, an optimal assessment of CNV lesions is ideally based on all three methods in conjunction, with OCT providing exact *in vivo* CNV volume measurements, histology giving high resolution structural insight and FA allowing for a functional readout of vessel permeability.
